# Supplementary material for: MetaRibo-Seq measures translation in microbiomes
Source: Nat Commun. 2020 Jun 29;11:3268. doi: 10.1038/s41467-020-17081-z (PMC7324362; doi:10.1038/s41467-020-17081-z)
Supplement: Supplementary file 10 — Supplementary Data 7 [file 41467_2020_17081_MOESM10_ESM.zip › File2/Confidence_High_Taxonomy/359405_out.krona.html]

Javascript must be enabled to view this page.

members
magnitude
magnitudeUnassigned
count
unassigned
taxon
rank

359405\_out

8

2
superkingdom
8

8
phylum
976

8
class
200643

8
order
171549

815
family
8

genus
816
8

8
817
species

SRS015854\_contig\_number\_18354SRS016095\_contig\_number\_18563SRS017247\_contig\_number\_8664SRS018656\_contig\_number\_11230SRS024075\_contig\_number\_2061SRS075878\_contig\_number\_2287SRS098644\_contig\_number\_9952SRS143598\_contig\_number\_11840
